# Deep sea sediments associated with cold seeps are a subsurface reservoir of viral diversity

**DOI:** 10.1038/s41396-021-00932-y

**Published:** 2021-03-01

**Authors:** Zexin Li, Donald Pan, Guangshan Wei, Weiling Pi, Chuwen Zhang, Jiang-Hai Wang, Yongyi Peng, Lu Zhang, Yong Wang, Casey R. J. Hubert, Xiyang Dong

**Affiliations:** 1 School of Marine Sciences, Sun Yat-Sen University, Zhuhai, China; 2 Department of Ecology and Environmental Studies, The Water School, Florida Gulf Coast University, Fort Myers, FL USA; 3 Key Laboratory of Marine Genetic Resources, Third Institute of Oceanography, Ministry of Natural Resources, Xiamen, China; 4 Key Laboratory of Coastal Environment and Resources of Zhejiang Province, School of Engineering, Westlake University, Hangzhou, China; 5 Institute of Advanced Technology, Westlake Institute for Advanced Study, Hangzhou, China; 6 Department of Life Science, Institute of Deep-sea Science and Engineering, Chinese Academy of Sciences, Sanya, China; 7 Department of Biological Sciences, University of Calgary, Calgary, AB Canada

**Keywords:** Environmental microbiology, Microbial ecology

## Abstract

In marine ecosystems, viruses exert control on the composition and metabolism of microbial communities, influencing overall biogeochemical cycling. Deep sea sediments associated with cold seeps are known to host taxonomically diverse microbial communities, but little is known about viruses infecting these microorganisms. Here, we probed metagenomes from seven geographically diverse cold seeps across global oceans to assess viral diversity, virus–host interaction, and virus-encoded auxiliary metabolic genes (AMGs). Gene-sharing network comparisons with viruses inhabiting other ecosystems reveal that cold seep sediments harbour considerable unexplored viral diversity. Most cold seep viruses display high degrees of endemism with seep fluid flux being one of the main drivers of viral community composition. In silico predictions linked 14.2% of the viruses to microbial host populations with many belonging to poorly understood candidate bacterial and archaeal phyla. Lysis was predicted to be a predominant viral lifestyle based on lineage-specific virus/host abundance ratios. Metabolic predictions of prokaryotic host genomes and viral AMGs suggest that viruses influence microbial hydrocarbon biodegradation at cold seeps, as well as other carbon, sulfur and nitrogen cycling via virus-induced mortality and/or metabolic augmentation. Overall, these findings reveal the global diversity and biogeography of cold seep viruses and indicate how viruses may manipulate seep microbial ecology and biogeochemistry.

## Introduction

Marine cold seeps are typically found at the edges of continental shelves and feature mainly gaseous and liquid hydrocarbons from deep geologic sources [1, 2]. Seep fluids can be produced through biological processes (methanogenesis in shallow sediments) or derive from thermogenic oil and gas systems that have been present for long periods of time in more deeply buried strata [3, 4]. In the context of global climate change, methane and other short-chain alkanes escaping from deep sea cold seep sediments can reach the atmosphere, exacerbating the greenhouse effect [5]. Understanding biogeochemical cycling in marine sediments associated with cold seeps is thus important for meeting critical energy and climate challenges.

Cold seeps are chemosynthetic ecosystems and contain an extensive diversity of archaea and bacteria which play important roles in hydrocarbon metabolism [6, 7]. These microbial populations are not only highly active in influencing seep biogeochemistry at the sediment-water interface [8], but also contribute to a variety of biological processes such as sulfate reduction, sulfur oxidation, denitrification, metal reduction and methanogenesis within the seabed [2, 8]. Viruses have also been observed in cold seep sediments. Epifluorescence microscopy of sediments from the Gulf of Mexico revealed that viral-like particle counts and virus-to-prokaryote ratios at cold seeps were significantly higher than in surrounding sediments, suggesting that these habitats may be hot spots for viruses [9]. This agrees with the elevated microbial activity at cold seeps driven by the availability of energy-rich substrates supplied from below. In addition, novel viruses have also been discovered in methane seep sediments, including a novel sister clade to the *Microvirus* genus of *Enterobacteria* phage and a putative archaeal virus linked to an anaerobic methane-oxidizing (ANME) clade [10, 11]. These findings suggest that cold seeps harbour abundant and undiscovered viruses potentially influencing their microbial hosts and consequently, biogeochemical cycling at cold seeps.

Knowledge of the ecological roles of viruses in deep sea sediments has been limited by difficulties in sampling and extracting viral particles (virions) from sediments [12]. In recent years, developments in sequencing and bioinformatics have enabled the analysis of viruses recovered from metagenomes sequenced without prior virion separation. These methods have greatly advanced viral ecology from the identification of novel viruses to the global distribution of viruses. Studies from a variety of environments such as thawing permafrost [13], mangroves [14], arctic lakes [15], freshwater lakes [16], deep sea sediments [17], the terrestrial subsurface [18, 19], and especially seawater [20–22] have suggested that prokaryotic viruses act as key agents in natural ecosystems via a range of interactions with their microbial hosts. Viruses can influence organic carbon and nutrient turnover by top-down control of microbial abundance via lysis of cells and subsequent release of cellular contents during lytic infection [23]. They can also reprogramme host metabolism through horizontal gene transfer or via auxiliary metabolic genes (AMGs) in their genomes that are expressed during infection. In peatland soils along a permafrost thaw gradient in Sweden, virus-encoded glycoside hydrolases were found to play a role in complex carbon degradation [13]. In freshwater lakes fed with sediment-derived methane, some viruses were found to encode subunits of particulate methane monooxygenase, suggesting that they may augment bacterial aerobic methane oxidation during infection [24]. Many studies have also revealed the presence and abundance of viruses in deep sea sediments [12, 17, 25–30], and thus deep sea sediments associated with cold seeps present a unique opportunity to study viruses and their interactions with hosts in a chemosynthetic ecosystem often dominated by anaerobic methane oxidation. Several metagenomic sequencing efforts have been undertaken on cold seep sediments [2], yet most studies have focused exclusively on genomes of bacteria and archaea, neglecting the viruses which are also present.

In this study, we sought to expand the understanding of viral diversity and the ecological role of viruses in deep sea sediments associated with cold seeps. To this end, 28 publicly available marine sediment metagenomes from seven cold seeps around the world were analyzed to recover genomes of viruses in cold seep communities. Characterizing these viral communities enabled host predictions and identification of AMGs. Our findings reveal the global diversity and biogeography of seep viruses and their role in benthic microbial ecology and biogeochemistry.

## Methods

### Collection of metagenomic data sets for deep sea cold seeps

**Fig. 1:**
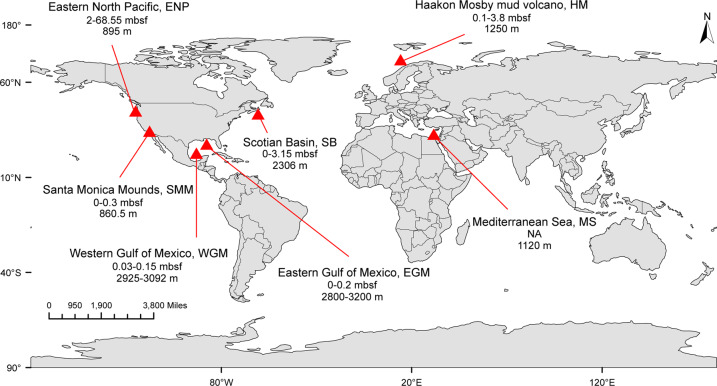
Geographic distribution of sampling sites where metagenomic data were collected. Locations of cold seep sites indicating the site name and abbreviation, sampling depth range in metres below sea floor (mbsf) and water depth in metres below sea level (m).

Metagenomic data sets were compiled from 28 sediment samples collected from seven cold seep sites across the global oceans (Fig. 1). These sites were as follows: Haakon Mosby mud volcano (HM); Eastern North Pacific ODP site 1244 (ENP); Mediterranean Sea, Amon mud volcano (MS); Santa Monica Mounds (SMM); Eastern Gulf of Mexico (EGM); Scotian Basin (SB); and Western Gulf of Mexico (WGM) (Supplementary Table 1 and references therein). Except for EGM and SB, metagenomic data sets along with metadata were downloaded from NCBI Sequence Read Archive and NCBI BioSample databases (https://www.ncbi.nlm.nih.gov). Sample collection and DNA sequencing of samples from EGM and SB are described in detail elsewhere [31, 32].

### Taxonomic profiling of microbial communities

To explore the prokaryotic composition of each sample, 16S rRNA gene fragments (i.e., miTags) were extracted from metagenomic raw reads using the phyloFlash pipeline [33]. Extracted 16S miTags were mapped to the SILVA SSU rRNA reference database (v132) [34] and assigned with an approximate taxonomic affiliation (nearest taxonomic unit, NTU).

### Metagenomic assembly

Raw reads were quality-controlled by trimming primers and adaptors and filtering out artifacts and low-quality reads using the Read_QC module within the metaWRAP pipeline [35]. Quality-controlled reads from each metagenome were individually assembled using MEGAHIT v1.1.3 [36] (default parameters). Short contigs (<1000 bp) were removed.

### Generation of prokaryotic metagenome-assembled genomes

For each assembly, contigs were binned using the binning module (parameters: --maxbin2 --metabat1 --metabat2) and consolidated into a final bin set using the Bin_refinement module (parameters: -c 50 -x 10) within metaWRAP [35]. All the produced bin sets were aggregated and de-replicated at 95% average nucleotide identity (ANI) using dRep v2.3.2 (parameters: -comp 50 -con 10 -sa 0.95) [37], resulting in a total of 592 species-level metagenome-assembled genomes (MAGs). Taxonomy of each MAG was initially assigned using GTDB-Tk v0.3.3 [38] based on the Genome Taxonomy Database (GTDB, http://gtdb.ecogenomic.org) taxonomy R04-RS89 [39]. The results were further refined using maximum-likelihood phylogeny inferred from a concatenation of 120 bacterial or 122 archaeal marker genes produced by GTDB-Tk. Bacterial and archaeal trees were built using RAxML v8 [40] called as follows: raxmlHPC-HYBRID -f a -n result -s input -c 25 -N 100 -p 12345 -m PROTCATLG -x 12345. Genomes were finally classified using the naming system of the NCBI taxonomy [41].

### Identification of viral contigs

Viral contigs were recovered from metagenome assemblies using VirSorter v1.0.5 [42] and VirFinder v1.1 [43]. Only contigs ≥10 kb were retained, based on the following criteria [21]: (1) VirSorter categories 1, 2, 4 and 5; (2) VirFinder score ≥0.9 and *p* < 0.05; (3) both identified by VirSorter categories 1–6 and VirFinder score ≥0.7 and *p* < 0.05. The identified contigs from each assembly were then compiled and clustered at 95% nucleotide identity using CD-HIT v4.8.1 (parameters: -c 0.95 -d 400 -T 20 -M 20000 -n 5) [44], producing 2,885 viral OTUs (vOTUs). Viral contigs identified by (3) were further validated using “What the Phage” (WtP) [45] and VIBRANT (v1.2.1, virome mode) [46], both with default settings. WtP v1.0.0 combines 12 established viral identification tools for the identification and analysis of viral sequences. These may represent a mixture of free viruses, proviruses and/or actively infecting viruses [13]. Completeness of viral genomes was estimated using the CheckV v0.6.0 pipeline [47]. CheckV v0.6.0 and VIBRANT v1.2.1 [46] were used to infer temperate lifestyles by identifying viral contigs that contain provirus integration sites or integrase genes. Lifestyles of other vOTUs were considered undetermined. Supplementary Fig. 1 shows an overview of the bioinformatic workflow used for the identification of viral populations.

### Comparisons to viral sequences from other environments and data sets

To place the 2,885 vOTUs in broader context, they were compared to viral contigs in public databases: (i) GOV 2.0 seawater [21] (*n* = 195,728); (ii) wetland sediment [48] (*n* = 1,212); (iii) Stordalen thawing permafrost [13] (*n* = 1,896). For each viral contig, open reading frames (ORFs) were called using Prodigal v2.6.3 [49], and the predicted protein sequences were used as input for vConTACT2 [50]. We followed the protocol published in protocols.io (https://www.protocols.io/view/applying-vcontact-to-viral-sequences-and-visualizi-x5xfq7n) for the application of vConTACT2 and visualization of the gene-sharing network in Cytoscape v3.7.2 [51] (edge-weighted spring-embedded model). Viral RefSeq (v85) was selected as the reference database, and Diamond was used for the protein–protein similarity method. Other parameters were set as default.

In order to determine overlaps between cold seep vOTU representatives and viral populations in the IMG/VR v3 dataset [52], we used rapid genome clustering to identify our vOTUs that share 95% identity with IMG/VR v3 populations [53] based on the scripts provided in CheckV [47]. The supporting code can be found at https://bitbucket.org/berkeleylab/checkv/src/master/.

### Viral taxonomic assignments

To identify the taxonomic affiliations of the vOTUs, ORFs predicated from Prodigal v2.6.3 were aligned against the NCBI Viral RefSeq v94 database using BLASTp (*E-*value of <0.0001, bit score ≥50) [14, 21, 54]. The BLASTp output was then imported into MEGAN v6.17.0 using the Lowest Common Ancestor algorithm for taxonomic analysis [55].

### Abundance profiles

RPKM (Reads per kilobase per million mapped reads) values were used to represent relative abundances of viruses and microorganisms. To calculate the RPKM values of each viral contig or MAG, quality-controlled reads from each sample were mapped to a viral contig database or to contigs compiled from the 592 MAGs with BamM v1.7.3 ‘make’ (https://github.com/Ecogenomics/BamM). Low-quality mappings were removed with BamM v1.7.3 ‘filter’ (parameters: --percentage_id 0.95 --percentage_aln 0.75). Filtered bam files were then passed to CoverM v0.3.1 (https://github.com/wwood/CoverM) to generate coverage profiles across samples (parameters: contig mode for viral contigs, genome mode for MAGs, --trim-min 0.10 --trim-max 0.90 --min-read-percent-identity 0.95 --min-read-aligned-percent 0.75 -m rpkm).

### Virus–host prediction

Four different in silico methods [13, 48, 56] were used to predict virus–host interactions. (1) *Nucleotide sequence homology*. Sequences of vOTUs and prokaryotic MAGs were compared using BLASTn. Match criteria were ≥75% coverage over the length of the viral contig, ≥70% minimum nucleotide identity, ≥50 bit score, and ≤0.001 *e*-value. (2) *Oligonucleotide frequency (ONF)*. VirHostMatcher v1.0 [57] was run with default parameters, with d_2_^*^ values ≤0.2 being considered as a match. (3) *Transfer RNA (tRNA) match*. Identification of tRNAs from prokaryotic MAGs and vOTUs was performed with ARAGORN v1.265 using the ‘-t’ option [58]. Match requirements were ≥90% length identity in ≥90% of the sequences by BLASTn [22]. (4) *CRISPR spacer match*. CRISPR arrays were assembled from quality-controlled reads using crass v1.0.1 with default parameters [59]. CRISPR spacers were then matched against viral contigs with ≤1 mismatch over the complete length of the spacer using BLASTn. For each matching CRISPR spacer, the repeat from the same assembled CRISPR array was compared against the prokaryotic MAGs using BLASTn with the same parameters, creating a virus–host link. Among potential linkages, *cas* genes of putative microbial hosts were inspected further using MetaErg v1.2.2 [60]. Only hits with adjacent *cas* genes were regarded as highly confident signals.

Whenever multiple hosts for a vOTU were predicted, the virus–host linkage supported by multiple approaches was chosen. Otherwise, to identify a single predicted host for each viral population, hosts were predicted using previously-reported [13] ranked criteria: (1) CRISPR spacer match with adjacent *cas* gene; (2) CRISPR spacer match without adjacent *cas* gene; (3) tRNA match or nucleotide sequence homology; (4) ONF comparison. The host predicted by the highest ranking criterion was chosen.

### Functional annotations of MAGs

Each MAG was first annotated using MetaErg v1.2.2 [60]. Predicted amino acid sequences were then used as queries for identification of key metabolic markers via METABOLIC v2.0 [61]. For phylogenetic analysis of McrA and DsrA, amino acid sequences were aligned using the MUSCLE algorithm [62] included in MEGA X [63]. All positions with less than 95% site coverage were eliminated. The maximum-likelihood phylogenetic tree was constructed in MEGA X using the JTT matrix-based model. The tree was bootstrapped with 50 replicates and midpoint-rooted.

### Identification of auxiliary metabolic genes

Prior to annotation, CheckV [47] was used to detect provirus boundaries and remove contamination from host-derived sequences. Annotations were performed mainly based on DRAM-v, the viral mode of DRAM [64]. DRAM-v requires output produced by VirSorter (e.g. VirSorter2) [65]. We first ran all the identified 2,885 vOTUs through VirSorter2 (--prep-for-dramv) to produce the affi-contigs.tab file and then annotated with the default databases. This process excluded 389 vOTUs owning to low viral scores based on VirSorter2, with 2,496 vOTUs being retained for AMG analysis. Putative AMGs with both auxiliary scores <4 and gene descriptions were selected according to the distillation output of DRAM-v annotations using default parameters [64, 66]. Following the method reported by Horst et al. [67], and to be conservative, we performed manual curation of the annotation output to improve the confidence in AMG identification, mainly by removing categories of genes related to nucleotide metabolism, organic nitrogen, glycosyl transferases and ribosomal proteins. Conserved domains of selected AMGs were then identified using NCBI CD-search tool [68]. Protein structural homology searches were performed using the Phyre2 web portal [69]. Genome maps for each contig encoding AMGs were visualized based on DRAM-v and VirSorter2 annotations. For comparison, VIBRANT [46] annotations were also performed on viral contigs based on KEGG, Pfam and VOG using default parameters. KEGG annotations categorized into “metabolic pathways” and “sulfur relay system” were reported as potential AMGs [46]. No curations were performed on the VIBRANT output.

### Statistical analyses

All statistical analyses were performed in R version 3.6.3. Alpha and beta diversity of viral communities were calculated using vegan package v2.5–6 [70]. Shapiro–Wilk and Bartlett’s tests were employed to test data normality and homoscedasticity prior to other statistical analysis. Non-metric multidimensional scaling (NMDS) was used to reduce dimensionality using the function capscale, based on Bray–Curtis dissimilarities generated from OTU tables with viral abundances (RPKM) using the vegdist function. The groupings of cold seep sites into different types [2] (mineral-prone vs mud-prone) and different sample depths (shallow vs deep) were individually verified using Analysis of Similarity (ANOSIM). For comparison of different cold seep sites, Shannon index was tested using Analysis of Variance (ANOVA) while Simpson and Chao1 indices were tested using a Kruskal–Wallis nonparametric test. For comparison of different cold seep systems and sample depths, Shannon index was tested using Student’s *T* test while Simpson and Chao1 indices were tested using Wilcoxon signed-rank test. Pearson correlations were calculated using the cor function.

## Results and discussion

To investigate the diversity and ecological function of viruses inhabiting cold seep sediments, a 0.38 Tbp compilation of metagenomic data was recruited from public databases and analyzed (Supplementary Table 1). Metagenomes were sequenced from 28 sediment samples obtained at seven seabed cold seeps across the global oceans, encompassing gas hydrates, oil and gas seeps, mud volcanoes and asphalt volcanoes (Fig. 1).

### Overview of bacterial and archaeal communities

To assess the overall microbial community structure in these sediments, 16S miTags were extracted from metagenomic reads for taxonomic profiling [33]. Classification of 16S miTags at the phylum level (class level for Proteobacteria) revealed the dominant bacterial lineages to be Chloroflexi (on average 23% of bacterial 16S miTags from 28 samples), Atribacteria (23%), *Gammaproteobacteria* (9%), *Deltaproteobacteria* (9%), and Planctomycetes (6%) (Supplementary Fig. 2). In shallow sediments (<0.2 m below the sea floor; mbsf), *Gammaproteobacteria* and *Deltaproteobacteria* were present in higher relative abundance, whereas Atribacteria and Chloroflexi predominated in deeper sediments that made up the majority of the sample set. For archaeal lineages, members from *Methanomicrobia* (phylum Euryarchaeota) were on average 30% of archaeal miTags, followed by Bathyarchaeota (TACK group) at 18%, and Lokiarchaeota (Asgard group) at 16% (Supplementary Fig. 3).

Assembly and binning of metagenomes resulted in 592 high- or medium-quality [71] microbial MAGs clustering at 95% ANI, nominally representing species-level groups [72]. These 460 bacterial and 132 archaeal MAGs spanned 46 known and four unclassified phyla (Supplementary Table 2). Within the domain Bacteria, members of Chloroflexi (*n* = 119 MAGs), *Deltaproteobacteria* (*n* = 67) and Planctomycetes (*n* = 44) were highly represented. Within the domain Archaea, MAGs were mainly affiliated with *Methanomicrobia* (*n* = 41), Bathyarchaeota (*n* = 21) and Lokiarchaeota (*n* = 18). Based on the read coverage of MAGs among the samples, the relative abundance of MAGs at the phylum level (class level for Proteobacteria and Euryarchaeota) was more similar between samples from the same region or depth, with distribution patterns being associated with cold seep types (Supplementary Fig. 4). No single MAG was found to be present in all seven cold seeps (Supplementary Table 3). All seven regions harboured MAGs belonging to *Deltaproteobacteria* (*n* = 67), Planctomycetes (*n* = 44), WOR-3 (*n* = 15), Bacteroidetes (*n* = 14), Heimdallarchaeota (*n* = 12) and Atribacteria (*n* = 11).

### Viruses from cold seep sediments are diverse and novel

From the 28 bulk shotgun metagenomes, 39,154 putative viral sequences were obtained, manually filtered and clustered at 95% ANI to represent approximately species-level taxonomy [21, 53]. This gave rise to 2,885 non-redundant cold seep vOTUs, each represented by contigs ≥10 kb in size, including four that were ≥200 kb (Supplementary Table 4) possibly corresponding to huge viruses [73]. Completeness of metagenome-assembled viral genomes or genome fragments was estimated using CheckV [47], giving rise to four different quality tiers: complete genomes (10% of the vOTUs), high-quality (4%), medium-quality (11%), and low-quality (58%), with the remainder being undetermined (Supplementary Table 4 and Supplementary Fig. 5).

**Fig. 2:**
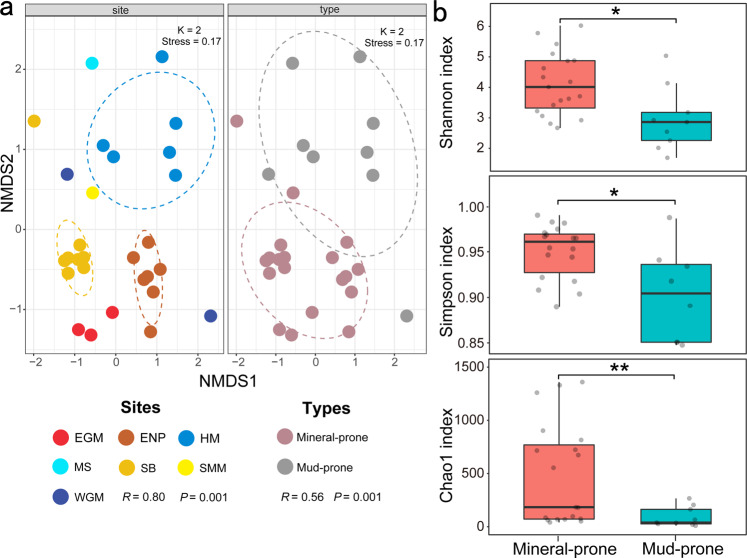
Comparison of viral community diversity between mineral-prone and mud-prone cold seeps. **a** NMDS analysis of a Bray-Curtis dissimilarity matrix calculated from RPKM values of vOTUs. ANOSIM was applied to test the difference in viral communities between different sites and different systems (mineral-prone vs mud-prone). **b** Shannon, Simpson and Chao1 indices of the viral community diversity from mineral-prone and mud-prone cold seeps. Asterisks denote significance, with * indicating *p* < 0.05, and ** indicating *p* < 0.01.

Though some cold seep vOTUs were very abundant in multiple sediment samples, a large majority (84%) of vOTUs were only present within a single cold seep site (Supplementary Table 5). Further analysis of viral distribution across the seven cold seep sites (ANOSIM, *R* = 0.80, *p* = 0.001; Fig. 2a) also shows that cold seep viruses display a high degree of endemism, similar to what was found previously in methane seep prokaryotic communities [74]. Viral Shannon diversity, Simpson diversity, and Chao1 richness were all observed to be significantly different (*p* < 0.05) between the seven sites (Supplementary Table 6). To arrange samples into environmentally meaningful groups, the seven cold seeps were designated as mineral-prone or mud-prone systems according to their fluid flow regime [2]. Mineral-prone systems have longer geologic history with slower emission of fluids, e.g., gas hydrates (ENP, SMM and SB) and oil and gas seeps (EGM), whereas younger mud-prone systems are high-flux, such as mud volcanoes (HM and MS), asphalt volcanoes (WGM), brine pools and brine basins. NMDS analysis revealed clear dissimilarity in viral communities between mineral-prone and mud-prone systems (ANOSIM, *R* = 0.56, *p* = 0.001; Fig. 2a). For the most part, viral communities from mineral-prone systems clustered together, except that SB_0 (i.e. surface sediment from 0.0 mbsf) deviated from other Scotian Basin viral communities as well as those from other mineral-prone seeps. Other factors also contribute to the structuring of the viral community, possibly including sediment depth (ANOSIM, *R* = 0.20, *p* = 0.01; Supplementary Fig. 6a). Shannon diversity, Simpson diversity, and Chao1 richness of viral communities were significantly higher in mineral-prone seep systems than in mud-prone (Fig. 2b), whereas no clear differences were observed between shallow (0–0.2 mbsf) and deep (>0.2 mbsf) groups (Supplementary Table 6 and Supplementary Fig. 6b). Overall, these results suggest that fluid flux explains a significant portion of the variance in viral diversity in cold seep sediments.

**Fig. 3:**
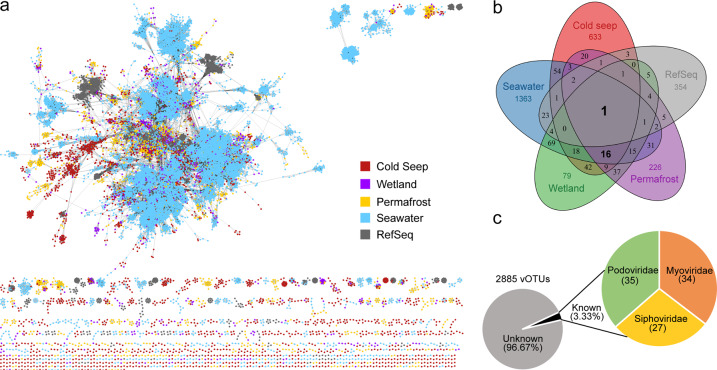
Taxonomic diversity of cold seep viruses. **a** Gene-sharing network of viral sequence space based on assembled viral genomes from cold seep sediment, wetland, permafrost, seawater and RefSeq prokaryotic viral genomes. Nodes represent viral genomes and edges indicate similarity based on shared protein clusters. **b** Venn diagram of shared viral clusters among the four environmental virus data sets and RefSeq. **c** Taxonomic assignments of vOTUs.

To investigate the relationship between cold seep vOTUs and publicly available virus sequences from a broader diversity of ecosystems, a gene-sharing network was constructed using vConTACT2 [50]. Such a weighted network can assign sequences into viral clusters (VCs) at approximately the genus level. Cold seep sediments, seawater, wetland, and permafrost vOTUs were grouped into 3,082 VCs (Fig. 3a and Supplementary Table 7). Only 17 VCs were shared amongst all ecosystems (Fig. 3b). The limited extent of clustering between viral genomes sampled from the various ecosystems may reflect a high degree of habitat specificity for viruses. Among cold seep sediment viruses, 1,742 out of 2,885 vOTUs were clustered into 804 VCs, with the majority (78.7%) being not encountered in any other ecosystem. This suggests that most cold seep viruses may be endemic to cold seeps (Fig. 3b and Supplementary Table 8). Among the 2,885 cold seep vOTUs, only 162 clustered with wetland-derived vOTUs, 154 with seawater-derived vOTUs, and 95 with permafrost-derived vOTUs (Supplementary Table 8). Very few cold seep viral vOTUs (~0.7%) clustered with taxonomically known genomes from Viral RefSeq (Fig. 3a and Supplementary Table 4), and the proportion is much lower in comparison with recent estimates in soil viruses using a similar approach [75]. Similarly, attempted taxonomic assignment of cold seep vOTUs using whole genome comparisons against 2,616 known bacterial and archaeal viruses from NCBI RefSeq (version 94) left >96% unclassified. The remainder were assigned to the *Caudovirales* order, specifically *Podoviradae* (*n* = 35), *Myoviradae* (*n* = 34) and *Siphoviradae* (*n* = 27) (Fig. 3c and Supplementary Table 4). These analyses show that cold seep sediments harbour considerable unexplored viral diversity. In addition, using rapid genome clustering (95% identity), only 62 vOTUs from our study were found to cluster with IMG/VR v3 populations (Supplementary Table 9), further suggesting that most cold seep vOTUs are endemic and unique compared to viruses in other ecosystems. The clustered vOTUs in the IMG/VR v3 database include 839 viral genomes, which are primarily derived from marine (54%) and human-associated (11%) samples.

### Virus–host linkages and host-linked viral abundance and viral lifestyles

**Fig. 4:**
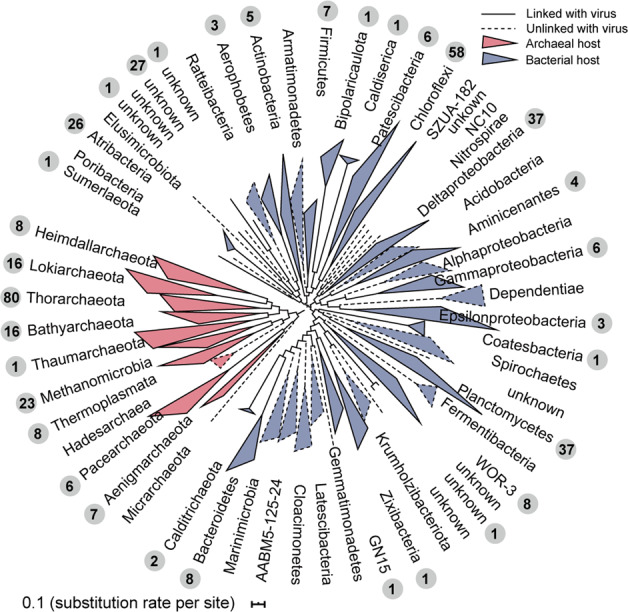
Cold seep virus–host linkages. Maximum-likelihood phylogenetic trees of bacterial and archaeal MAGs at the phylum level (class level for Proteobacteria and Euryarchaeota) were inferred from a concatenated alignment of 120 bacterial or 122 archaeal single-copy marker genes. Clades outlined by solid lines represent lineages predicted to include a host for one or more viral OTUs. The grey circle shows the number of vOTUs predicted to have a host in the clade.

Comparing sequence similarity, oligonucleotide frequencies, tRNA sequences and CRISPR spacers [76], putative hosts were predicted for 14.2% of the 2,885 cold seep vOTUs (Supplementary Table 10). Consistent with previous observations [76, 77], most of these vOTUs were predicted to have narrow host ranges, with only 54 vOTUs potentially exhibiting a broader host range across several phyla. Twenty-six vOTUs were linked to both bacterial and archaeal hosts, suggesting existence of viral infection across domains (Supplementary Table 10). A single predicted host was retained for each viral population (see “Methods”). Predicted prokaryotic hosts spanned 9 archaeal and 23 bacterial phyla, with Thorarchaeota (19% of virus-host pairs) and Chloroflexi (14%) being the most frequently predicted (Fig. 4). A considerable proportion (40%) of cold seep vOTUs were linked to archaea, including members of Bathyarchaeota, the Asgard group, *Methanomicrobia*, Thaumarchaeota and *Thermoplasmata*. Such broad ranges for archaeal viruses have not been reported previously in natural systems [13, 76]. As compared to the IMG/VR v3 database [52], several novel viral-bacterial linkages were also identified, including putative viruses for Bipolaricaulota, Coatesbacteria and Sumerlaeota. Based on the presence of functional marker genes within MAGs, predicted hosts include two aerobic methanotrophic *Methylococcales* (two vOTUs, Supplementary Tables 11), 13 anaerobic methane-oxidizing archaea (e.g. ANME-1 and ANME-2, Supplementary Fig. 7a), one non-methane multi-carbon alkane oxidizer within *Methanosarcinales* (Supplementary Fig. 7a), 16 sulfate reducers mostly belonging to *Deltaproteobacteria* (52 vOTUs, Supplementary Fig. 7b), and numerous respiring and fermentative heterotrophs (Supplementary Table 11). In total, we found 22 vOTUs that might infect archaea (Class *Methanomicrobia*) capable of anaerobic gaseous alkane oxidation based on methyl/alkyl-coenzyme M reductase (Supplementary Table 10). Along with the similar discovery of viruses that infect ANME clades in other methane seep sediments, e.g., the Nyegga methane seep and Coal Oil Point hydrocarbon seep [11], potential viruses infecting anaerobic gaseous alkane oxidizers are possibly widespread in cold seeps, in agreement with the dominance of these archaea [2]. The genome of the sulfate reducer *Desulfobacterales* 8_GM_sbin_oily_21 also harboured genes possibly encoding akyl-/arylalkylsuccinate synthases related to anaerobic degradation of longer alkanes and aromatic hydrocarbons. These results suggest that populations mediating hydrocarbon and sulfur cycling may be vulnerable to lysis by viral populations in cold seeps, where sulfate reduction is coupled to the anaerobic oxidation of methane and other seeping hydrocarbons. Predicted hosts were also identified within the candidate phyla radiation (six vOTUs were predicted to infect Patescibacteria) and DPANN archaea (13 vOTUs were predicted to infect Pacearchaeota or Aenigmarchaeota). Due to the limited metabolic capabilities and small cell sizes, many CPR and DPANN organisms are likely to be obligate symbionts of other bacteria and archaea [78]. The impact of viral infection on obligate symbionts and any consequences for the larger organisms hosting those symbionts are not yet known, although it has been suggested that they may protect those hosts from viral predation [78].

**Fig. 5:**
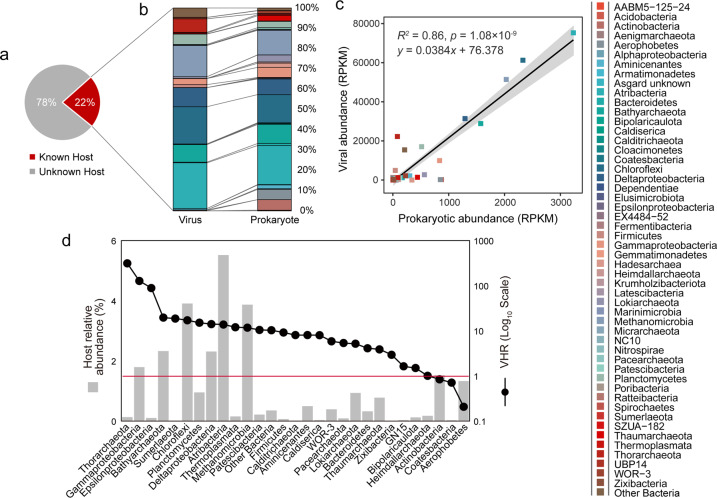
Relative abundance patterns of viruses and their predicted hosts in cold seep sediments. **a** Percentage of vOTUs based on relative abundance in which a host was predicted or not. **b** Relative abundances of vOTUs and their predicted hosts grouped by the host taxonomy. **c** Significant Pearson correlation between relative abundances of viruses and their hosts (calculated by normalized mean coverage depth, reads per kilobase mapped reads: RPKM). **d** Lineage-specific virus–host abundance ratios (VHR) for all predicted microbial hosts. The red line indicates a 1:1 ratio. Predicted hosts in (**b**) and (**c**) are indicated in the colour bar on the right side (colour figure online).

Based on abundances determined by read mapping, targeted hosts were predicted for >20% of the cold seep viral community (Fig. 5a). When grouped at the phylum level (class level for Proteobacteria and Euryarchaeota), the composition of predicted microbial hosts agreed well with that of their viruses (Fig. 5b). This is supported by regression modelling of the abundances of hosts and lineage-specific viruses (Fig. 5c). By applying metagenomic read recruitment, most viruses had higher genome coverage compared to their hosts, suggesting that most taxa may be undergoing active viral replication and possibly lysis at the time of sample collection [79]. Lineage-specific virus/host abundance ratios (i.e. VHR) for most taxa were greater than one with Thorarchaeota being the highest at 10^2.5^ (Fig. 5d), indicating a high level of active viral genome replication. This is in accordance with the presence of higher abundances of viral particles detected by epifluorescence microscopy in cold seep sediments compared to non-cold seep sediments in the Gulf of Mexico [9]. Thus, viral lysis may be a major top-down factor [80], contributing to significant microbial mortality in cold seep sediments. In addition, based on the presence of integrase genes and/or being located within their host genomes, at least 372 cold seep vOTUs were predicted to be lysogenic (i.e., temperate viruses, Supplementary Fig. 8 and Supplementary Table 4).

### Viral AMGs involved in carbon, sulfur and nitrogen transformations

**Fig. 6:**
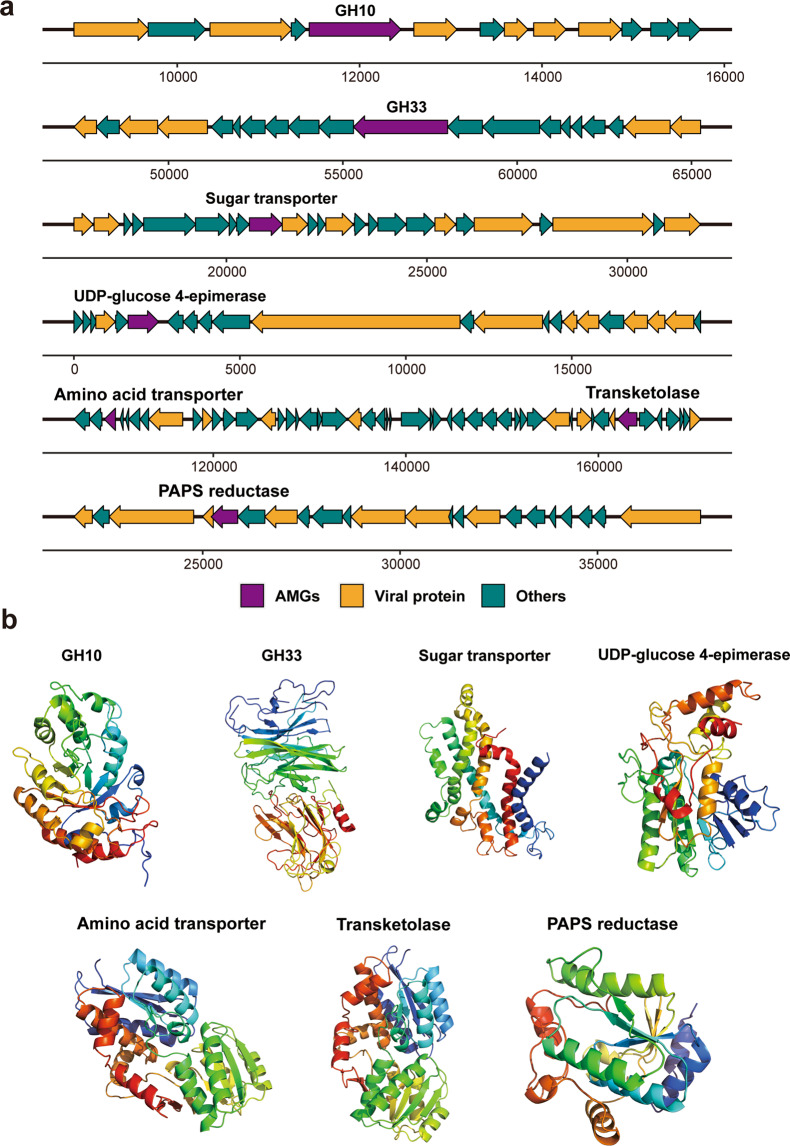
Genomic context and protein structure of selected virus-encoded auxiliary metabolic genes. **a** Genome map of representative AMG-encoding viruses, with the AMGs of interest in purple, virus-related genes in orange, and non-phage-like or uncharacterized genes in teal. Detailed annotations can be found in Supplementary Table 14. **b** Tertiary structures of selected AMGs based on structural modelling using Phyre2 (colour figure online).

To further understand how viruses might affect the biogeochemistry of cold seep sediments, viral contigs encoding AMGs that supplement host metabolism during infection were examined. Overall, cold seep viruses tend to encode AMGs for cofactor/vitamin and carbohydrate metabolism based on VIBRANT [46] annotations (Supplementary Table 12). A significant portion also encoded AMGs for amino acid and glycan metabolism. Based on DRAM-v annotations [64] and manual curation [67], we identified 32 AMG genes from 29 vOTUs encoding carbohydrate-active enzymes involved in the initial breakdown of complex carbohydrates (Supplementary Table 13). Six of these genes were affiliated with glycoside hydrolases predicted to catalyze hydrolysis of complex sugars based on modelling of protein three-dimensional structures (Fig. 6 and Supplementary Table 13). These six bacteria- or archaea-like viral glycoside hydrolases spanned four families, including GH10, GH136, GH33 and GH74. They were found in five vOTUs from four different VCs with hosts being unidentified (Supplementary Tables 7, 10 and 13). In addition, AMGs related to ABC transporters for carbohydrate, central carbon (e.g., UDP-glucose 4-epimerase) and C1 metabolism (e.g., transketolase) were also identified (Fig. 6 and Supplementary Table 13). These genes have been increasingly recovered as putative AMGs in viruses, which may contribute to nucleotide and energy production during infection [75, 81, 82]. Deep sea sediment microorganisms associated with cold seeps were reported to be involved in a variety of carbohydrate degradation reactions for processing detrital organic matter supplied from the overlying water column [31, 32]. Infection by viruses containing genes related to carbohydrate degradation in general is consistent with their supportive role in host metabolism during the infection cycle [13].

Based on VIRBANT annotations (Supplementary Table 12), the most common AMG related to sulfur metabolism within the viral contigs was phosphoadenosine phosphosulfate reductase (PAPS reductase or CysH), which is predicted to participate in assimilatory sulfate reduction. Viral *cysH* has also been found in viral sequences obtained from oxygen-deficient water columns [83], rumen [84], a deep freshwater lake [16] and sulfidic mine tailings [85]. Here, we manually confirmed that three vOTUs from two distinct viral genera encoded PAPS reductase genes (Fig. 6a and Supplementary Table 13). The three representative PAPS reductase genes contain the conserved domain and structural configuration of PAPS reductase (Fig. 6b) that assimilates sulfates for biosynthesis of methionine and cysteine [83]. One of these three vOTUs was identified to potentially infect the ANME-1 clade. Cold seep sediments are in general considered to feature an abundance of organic matter but are nitrogen-limited for supporting biological biomass production [2, 86]. We suspect that viral-encoded PAPS reductase genes can enhance biosynthesis of amino acids during the proliferation of typical cold seep microorganisms like ANME archaea in seep sediments, supplementing the nitrogen fixation capacity of cold seep microbial populations [2, 87]. In addition, ABC transporters related to amino acids were also identified (Supplementary Table 13), highlighting another potential route for organic nitrogen metabolism in these communities.

## Conclusions

Due to the challenges of deep sea sediment sampling and laboratory cultivation of microbial communities along with their viruses, the roles that viruses play in influencing microbial mortality, ecology, and evolution remain largely unexplored in marine sediments associated with cold seeps [17, 88]. In this study, in-depth exploration of untargeted de novo metagenomic data successfully revealed novel, abundant, and diverse bacterial and archaeal viruses. Many of the putative microbial hosts for seep viruses belong to taxonomic groups with no cultured representatives. These results therefore expand the diversity of archaeal viruses, especially those infecting important archaeal lineages in hydrocarbon seep microbiomes, e.g., members of the Euryarchaeota, Bathyarchaeota, and the Asgard group. While a significant portion of the viruses appear to be lysogenic, the high read coverages for many viral genomes suggest that viral lysis is a major source of microbial mortality and biomass turnover in cold seep sediments. Virus-encoded AMGs, including genes related to carbon, sulfur, and nitrogen metabolism, may augment the metabolism of prokaryotic hosts during infection, potentially altering biogeochemical processes mediated by cold seep microorganisms. As subsurface reservoirs of prokaryotic diversity and hot spots of microbial activity, cold seeps additionally represent oases of viruses and viral activity. Much remains to be revealed about the contribution of viruses to the functioning of cold seeps and other marine environments, especially with respect to their potential role in horizontal gene transfer which was not addressed in this study. With only a fraction of vOTUs identified here able to be classified and many of them predicted to infect poorly characterized taxa, there remain large gaps in the understanding of the microbiology of these environments.

## Supplementary information

Supplementary Figures

Supplementary Tables

## Data Availability

Sequences of 2,885 viral contigs and 592 de-replicated metagenome-assembled genomes can be found at figshare (10.6084/m9.figshare.12922229). All other data are available from the corresponding author upon request.

## References

[1] Suess E (2014). Marine cold seeps and their manifestations: geological control, biogeochemical criteria and environmental conditions. Int J Earth Sci.

[2] Joye SB (2020). The geology and biogeochemistry of hydrocarbon seeps. Annu Rev Earth Planet Sci.

[3] Etiope G, Panieri G, Fattorini D, Regoli F, Vannoli P, Italiano F (2014). A thermogenic hydrocarbon seep in shallow Adriatic Sea (Italy): Gas origin, sediment contamination and benthic foraminifera. Mar Pet Geol.

[4] Kennicutt, MC Habitats and biota of the Gulf of Mexico: before the deepwater horizon oil spill. Ward CH, editor. New York, NY: Springer New York; 2017. p. 275–358.

[5] Ruppel CD, Kessler JD (2017). The interaction of climate change and methane hydrates. Rev Geophys.

[6] Kniemeyer O, Musat F, Sievert SM, Knittel K, Wilkes H, Blumenberg M (2007). Anaerobic oxidation of short-chain hydrocarbons by marine sulphate-reducing bacteria. Nature.

[7] Jaekel U, Musat N, Adam B, Kuypers M, Grundmann O, Musat F (2013). Anaerobic degradation of propane and butane by sulfate-reducing bacteria enriched from marine hydrocarbon cold seeps. ISME J.

[8] Teske A, Carvalho V. Marine hydrocarbon seeps: microbiology and biogeochemistry of a global marine habitat. Cham, Switzerland: Springer Nature; 2020.

[9] Kellogg CA (2010). Enumeration of viruses and prokaryotes in deep-sea sediments and cold seeps of the Gulf of Mexico. Deep Sea Res Part II Top Stud Oceanogr.

[10] Bryson SJ, Thurber AR, Correa AM, Orphan VJ, Vega Thurber R (2015). A novel sister clade to the enterobacteria microviruses (family Microviridae) identified in methane seep sediments. Environ Microbiol.

[11] Paul BG, Bagby SC, Czornyj E, Arambula D, Handa S, Sczyrba A (2015). Targeted diversity generation by intraterrestrial archaea and archaeal viruses. Nat Commun.

[12] Pan D, Morono Y, Inagaki F, Takai K (2019). An improved method for extracting viruses from sediment: detection of far more viruses in the subseafloor than previously reported. Front Microbiol.

[13] Emerson JB, Roux S, Brum JR, Bolduc B, Woodcroft BJ, Jang HB (2018). Host-linked soil viral ecology along a permafrost thaw gradient. Nat Microbiol.

[14] Jin M, Guo X, Zhang R, Qu W, Gao B, Zeng R (2019). Diversities and potential biogeochemical impacts of mangrove soil viruses. Microbiome.

[15] Labbe M, Girard C, Vincent WF, Culley AI (2020). Extreme viral partitioning in a marine-derived high arctic lake. mSphere.

[16] Okazaki Y, Nishimura Y, Yoshida T, Ogata H, Nakano SI (2019). Genome-resolved viral and cellular metagenomes revealed potential key virus-host interactions in a deep freshwater lake. Environ Microbiol.

[17] Backstrom D, Yutin N, Jorgensen SL, Dharamshi J, Homa F, Zaremba-Niedwiedzka K (2019). Virus genomes from deep sea sediments expand the ocean megavirome and support independent origins of viral gigantism. mBio.

[18] Daly RA, Roux S, Borton MA, Morgan DM, Johnston MD, Booker AE (2019). Viruses control dominant bacteria colonizing the terrestrial deep biosphere after hydraulic fracturing. Nat Microbiol.

[19] Daly RA, Borton MA, Wilkins MJ, Hoyt DW, Kountz DJ, Wolfe RA (2016). Microbial metabolisms in a 2.5-km-deep ecosystem created by hydraulic fracturing in shales. Nat Microbiol.

[20] Roux S, Brum JR, Dutilh BE, Sunagawa S, Duhaime MB, Loy A (2016). Ecogenomics and potential biogeochemical impacts of globally abundant ocean viruses. Nature.

[21] Gregory AC, Zayed AA, Conceicao-Neto N, Temperton B, Bolduc B, Alberti A (2019). Marine DNA viral macro- and microdiversity from pole to pole. Cell.

[22] Coutinho FH, Silveira CB, Gregoracci GB, Thompson CC, Edwards RA, Brussaard CPD (2017). Marine viruses discovered via metagenomics shed light on viral strategies throughout the oceans. Nat Commun.

[23] Breitbart M, Bonnain C, Malki K, Sawaya NA (2018). Phage puppet masters of the marine microbial realm. Nat Microbiol.

[24] Chen LX, Meheust R, Crits-Christoph A, McMahon KD, Nelson TC, Slater GF (2020). Large freshwater phages with the potential to augment aerobic methane oxidation. Nat Microbiol.

[25] Cai L, Jorgensen BB, Suttle CA, He M, Cragg BA, Jiao N (2019). Active and diverse viruses persist in the deep sub-seafloor sediments over thousands of years. ISME J.

[26] Danovaro R, Dell’Anno A, Corinaldesi C, Magagnini M, Noble R, Tamburini C (2008). Major viral impact on the functioning of benthic deep-sea ecosystems. Nature.

[27] Middelboe M, Glud RN, Wenzhöfer F, Oguri K, Kitazato H (2006). Spatial distribution and activity of viruses in the deep-sea sediments of Sagami Bay. Jpn Deep Sea Res Part 1 Oceanogr Res Pap.

[28] Danovaro R, Serresi M (2000). Viral density and virus-to-bacterium ratio in deep-sea sediments of the Eastern Mediterranean. Appl Environ Microbiol.

[29] Hewson I, Fuhrman JA (2003). Viriobenthos production and virioplankton sorptive scavenging by suspended sediment particles in coastal and pelagic waters. Micro Ecol.

[30] Corinaldesi C, Dell’Anno A, Danovaro R (2007). Viral infection plays a key role in extracellular DNA dynamics in marine anoxic systems. Limnol Oceanogr.

[31] Dong X, Greening C, Rattray JE, Chakraborty A, Chuvochina M, Mayumi D (2019). Metabolic potential of uncultured bacteria and archaea associated with petroleum seepage in deep-sea sediments. Nat Commun.

[32] Dong X, Rattray JE, Campbell DC, Webb J, Chakraborty A, Adebayo O (2020). Thermogenic hydrocarbon biodegradation by diverse depth-stratified microbial populations at a Scotian Basin cold seep. Nat Commun.

[33] Gruber-Vodicka HR, Seah BKB, Pruesse E (2020). phyloFlash: rapid small-subunit rRNA profiling and targeted assembly from metagenomes. mSystems.

[34] Quast C, Pruesse E, Yilmaz P, Gerken J, Schweer T, Yarza P (2013). The SILVA ribosomal RNA gene database project: improved data processing and web-based tools. Nucleic Acids Res.

[35] Uritskiy GV, DiRuggiero J, Taylor J (2018). MetaWRAP-a flexible pipeline for genome-resolved metagenomic data analysis. Microbiome.

[36] Li D, Luo R, Liu CM, Leung CM, Ting HF, Sadakane K (2016). MEGAHIT v1.0: A fast and scalable metagenome assembler driven by advanced methodologies and community practices. Methods.

[37] Olm MR, Brown CT, Brooks B, Banfield JF (2017). dRep: a tool for fast and accurate genomic comparisons that enables improved genome recovery from metagenomes through de-replication. ISME J.

[38] Chaumeil P-A, Mussig AJ, Hugenholtz P, Parks DH (2019). GTDB-Tk: a toolkit to classify genomes with the genome taxonomy database. Bioinformatics.

[39] Parks DH, Chuvochina M, Chaumeil PA, Rinke C, Mussig AJ, Hugenholtz P (2020). A complete domain-to-species taxonomy for Bacteria and Archaea. Nat Biotechnol.

[40] Stamatakis A (2014). RAxML version 8: a tool for phylogenetic analysis and post-analysis of large phylogenies. Bioinformatics.

[41] Federhen S (2012). The NCBI taxonomy database. Nucleic Acids Res.

[42] Roux S, Enault F, Hurwitz BL, Sullivan MB (2015). VirSorter: mining viral signal from microbial genomic data. PeerJ.

[43] Ren J, Ahlgren NA, Lu YY, Fuhrman JA, Sun F (2017). VirFinder: a novel k-mer based tool for identifying viral sequences from assembled metagenomic data. Microbiome.

[44] Fu L, Niu B, Zhu Z, Wu S, Li W (2012). CD-HIT: accelerated for clustering the next-generation sequencing data. Bioinformatics.

[45] Marquet M, Hölzer M, Pletz MW, Viehweger A, Makarewicz O, Ehricht R, et al. What the phage: a scalable workflow for the identification and analysis of phage sequences. 2020. https://www.biorxiv.org/content/10.1101/2020.07.24.219899v1. 10.1093/gigascience/giac110 PMC9673492 36399058

[46] Kieft K, Zhou Z, Anantharaman K (2020). VIBRANT: automated recovery, annotation and curation of microbial viruses, and evaluation of viral community function from genomic sequences. Microbiome.

[47] Nayfach S, Camargo AP, Schulz F, Eloe-Fadrosh E, Roux S, Kyrpides NC. CheckV assesses the quality and completeness of metagenome-assembled viral genomes. Nat Biotechnol. 2020. 10.1101/2020.1105.1106.081778. 10.1038/s41587-020-00774-7 PMC8116208 33349699

[48] Dalcin Martins P, Danczak RE, Roux S, Frank J, Borton MA, Wolfe RA (2018). Viral and metabolic controls on high rates of microbial sulfur and carbon cycling in wetland ecosystems. Microbiome.

[49] Hyatt D, Chen GL, Locascio PF, Land ML, Larimer FW, Hauser LJ (2010). Prodigal: prokaryotic gene recognition and translation initiation site identification. BMC Bioinform.

[50] Bin Jang H, Bolduc B, Zablocki O, Kuhn JH, Roux S, Adriaenssens EM (2019). Taxonomic assignment of uncultivated prokaryotic virus genomes is enabled by gene-sharing networks. Nat Biotechnol.

[51] Shannon P, Markiel A, Ozier O, Baliga NS, Wang JT, Ramage D (2003). Cytoscape: a software environment for integrated models of biomolecular interaction networks. Genome Res.

[52] Roux S, Paez-Espino D, Chen IA, Palaniappan K, Ratner A, Chu K, et al. IMG/VR v3: an integrated ecological and evolutionary framework for interrogating genomes of uncultivated viruses. Nucleic Acids Res. 2020;49:D764–75. 10.1093/nar/gkaa946 PMC7778971 33137183

[53] Roux S, Adriaenssens EM, Dutilh BE, Koonin EV, Kropinski AM, Krupovic M (2019). Minimum information about an uncultivated virus genome (MIUViG). Nat Biotechnol.

[54] Castelan-Sanchez HG, Lopez-Rosas I, Garcia-Suastegui WA, Peralta R, Dobson ADW, Batista-Garcia RA (2019). Extremophile deep-sea viral communities from hydrothermal vents: structural and functional analysis. Mar Genom.

[55] Huson DH, Auch AF, Qi J, Schuster SC (2007). MEGAN analysis of metagenomic data. Genome Res.

[56] Tominaga K, Morimoto D, Nishimura Y, Ogata H, Yoshida T (2020). In silico prediction of virus-host interactions for marine bacteroidetes with the use of metagenome-assembled genomes. Front Microbiol.

[57] Ahlgren NA, Ren J, Lu YY, Fuhrman JA, Sun F (2017). Alignment-free *d*_2_^*^oligonucleotide frequency dissimilarity measure improves prediction of hosts from metagenomically-derived viral sequences. Nucleic Acids Res.

[58] Laslett D, Canback B (2004). ARAGORN, a program to detect tRNA genes and tmRNA genes in nucleotide sequences. Nucleic Acids Res.

[59] Skennerton CT, Imelfort M, Tyson GW (2013). Crass: identification and reconstruction of CRISPR from unassembled metagenomic data. Nucleic Acids Res.

[60] Dong X, Strous M (2019). An integrated pipeline for annotation and visualization of metagenomic contigs. Front Genet.

[61] Zhou Z, Tran PQ, Breister AM, Liu Y, Kieft K, Cowley ES, et al. METABOLIC: a scalable high-throughput metabolic and biogeochemical functional trait profiler based on microbial genomes. 2020. https://www.biorxiv.org/content/10.1101/761643v1.

[62] Edgar RC (2004). MUSCLE: a multiple sequence alignment method with reduced time and space complexity. BMC Bioinform.

[63] Kumar S, Stecher G, Li M, Knyaz C, Tamura K (2018). MEGA X: molecular evolutionary genetics analysis across computing platforms. Mol Biol Evol.

[64] Shaffer M, Borton MA, McGivern BB, Zayed AA, La Rosa SL, Solden LM (2020). DRAM for distilling microbial metabolism to automate the curation of microbiome function. Nucleic Acids Res.

[65] Guo J, Bolduc B, Zayed AA, Varsani A, Dominguez-Huerta G, Delmont TO (2021). VirSorter2: a multi-classifier, expert-guided approach to detect diverse DNA and RNA viruses. Microbiome.

[66] Vik D, Gazitua MC, Sun CL, Zayed AA, Aldunate M, Mulholland MR et al. Genome-resolved viral ecology in a marine oxygen minimum zone. Environ Microbiol. 2020. 10.1111/1462-2920.15313. 10.1111/1462-2920.15313 33185964

[67] ter Horst AM, Santos-Medellin C, Sorensen JW, Zinke LA, Wilson RM, Johnston ER, et al. Minnesota peat viromes reveal terrestrial and aquatic niche partitioning for local and global viral populations. 2020. https://www.biorxiv.org/content/10.1101/2020.12.15.422944v1.full. 10.1186/s40168-021-01156-0 PMC8626947 34836550

[68] Lu S, Wang J, Chitsaz F, Derbyshire MK, Geer RC, Gonzales NR (2020). CDD/SPARCLE: the conserved domain database in 2020. Nucleic Acids Res.

[69] Kelley LA, Mezulis S, Yates CM, Wass MN, Sternberg MJ (2015). The Phyre2 web portal for protein modeling, prediction and analysis. Nat Protoc.

[70] Dixon P (2003). VEGAN, a package of R functions for community ecology. J Veg Sci.

[71] Bowers RM, Kyrpides NC, Stepanauskas R, Harmon-Smith M, Doud D, Reddy TBK (2017). Minimum information about a single amplified genome (MISAG) and a metagenome-assembled genome (MIMAG) of bacteria and archaea. Nat Biotechnol.

[72] Jain C, Rodriguez RL, Phillippy AM, Konstantinidis KT, Aluru S (2018). High throughput ANI analysis of 90K prokaryotic genomes reveals clear species boundaries. Nat Commun.

[73] Al-Shayeb B, Sachdeva R, Chen LX, Ward F, Munk P, Devoto A (2020). Clades of huge phages from across Earth’s ecosystems. Nature.

[74] Ruff SE, Biddle JF, Teske AP, Knittel K, Boetius A, Ramette A (2015). Global dispersion and local diversification of the methane seep microbiome. Proc Natl Acad Sci USA.

[75] Trubl G, Jang HB, Roux S, Emerson JB, Solonenko N, Vik DR (2018). Soil viruses are underexplored players in ecosystem carbon processing. mSystems.

[76] Paez-Espino D, Eloe-Fadrosh EA, Pavlopoulos GA, Thomas AD, Huntemann M, Mikhailova N (2016). Uncovering Earth’s virome. Nature.

[77] Roux S, Hallam SJ, Woyke T, Sullivan MB (2015). Viral dark matter and virus-host interactions resolved from publicly available microbial genomes. elife.

[78] Castelle CJ, Brown CT, Anantharaman K, Probst AJ, Huang RH, Banfield JF (2018). Biosynthetic capacity, metabolic variety and unusual biology in the CPR and DPANN radiations. Nat Rev Microbiol.

[79] Jarett JK, Dzunkova M, Schulz F, Roux S, Paez-Espino D, Eloe-Fadrosh E (2020). Insights into the dynamics between viruses and their hosts in a hot spring microbial mat. ISME J.

[80] Orsi WD (2018). Ecology and evolution of seafloor and subseafloor microbial communities. Nat Rev Microbiol.

[81] Hurwitz BL, Brum JR, Sullivan MB (2015). Depth-stratified functional and taxonomic niche specialization in the ‘core’ and ‘flexible’ Pacific Ocean Virome. ISME J.

[82] Brum JR, Sullivan MB (2015). Rising to the challenge: accelerated pace of discovery transforms marine virology. Nat Rev Microbiol.

[83] Mara P, Vik D, Pachiadaki MG, Suter EA, Poulos B, Taylor GT (2020). Viral elements and their potential influence on microbial processes along the permanently stratified Cariaco Basin redoxcline. ISME J.

[84] Anderson CL, Sullivan MB, Fernando SC (2017). Dietary energy drives the dynamic response of bovine rumen viral communities. Microbiome.

[85] Gao SM, Schippers A, Chen N, Yuan Y, Zhang MM, Li Q (2020). Depth-related variability in viral communities in highly stratified sulfidic mine tailings. Microbiome.

[86] Zhao R, Summers ZM, Christman GD, Yoshimura KM, Biddle JF (2020). Metagenomic views of microbial dynamics influenced by hydrocarbon seepage in sediments of the Gulf of Mexico. Sci Rep.

[87] Dekas AE, Poretsky RS, Orphan VJ (2009). Deep-sea archaea fix and share nitrogen in methane-consuming microbial consortia. Science.

[88] Zheng, X, Liu, W, Dai, X, Zhu, Y, Wang, J, Zhu, Y et al. Extraordinary diversity of viruses in deep-sea sediments as revealed by metagenomics without prior virion separation. Environ Microbiol. 2020. 10.1111/1462-2920.15154. 10.1111/1462-2920.15154 32627268

