# Supplementary material for: Deep sea sediments associated with cold seeps are a subsurface reservoir of viral diversity
Source: ISME J. 2021 Mar 1;15(8):2366–78. doi: 10.1038/s41396-021-00932-y (PMC8319345; doi:10.1038/s41396-021-00932-y)
Supplement: Supplementary file 1 — Supplementary Figures [file 41396_2021_932_MOESM1_ESM.pdf]

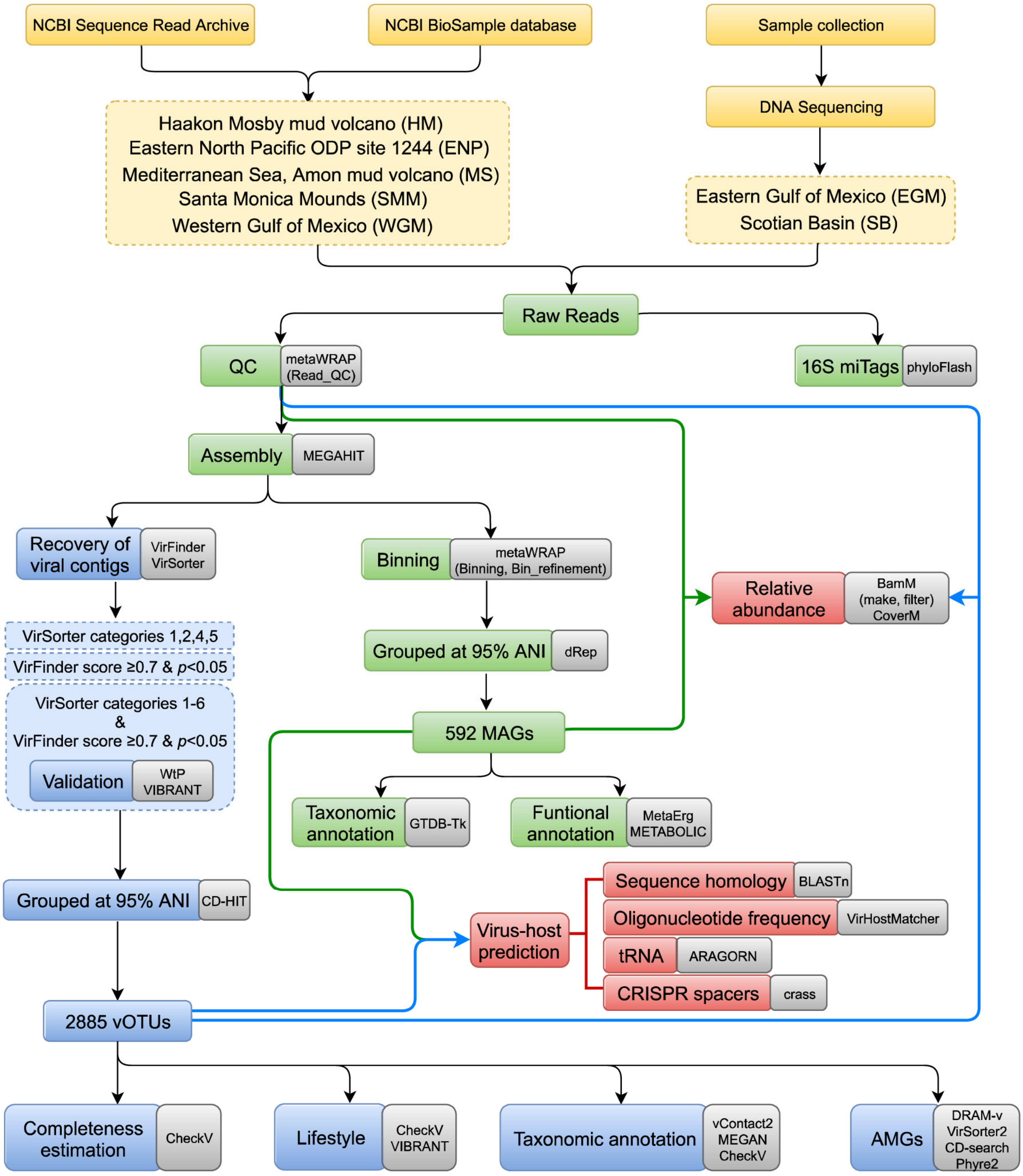

Supplementary Figure 1. The diagram showing the bioinformatic workflow for the identification of viral populations. Yellow: data collection; green: microbial analysis; blue: viral analysis; red: interactions between hosts and viruses; grey: software used.

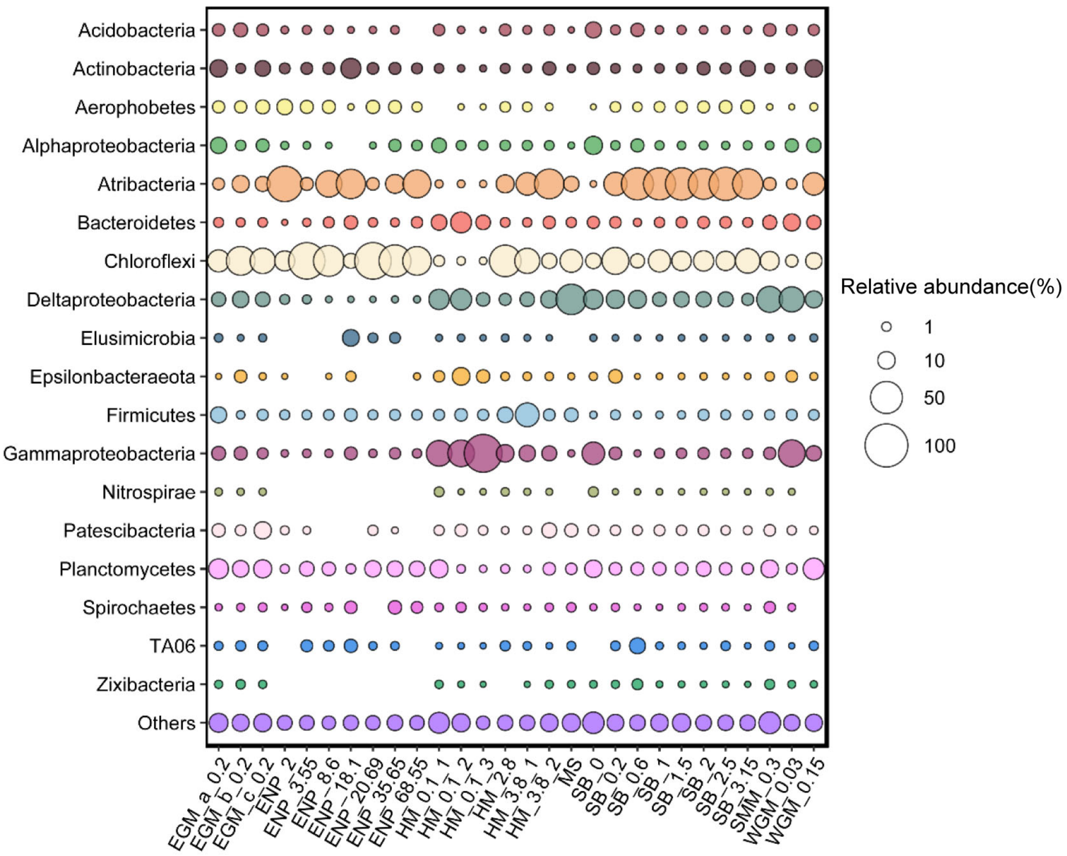

Supplementary Figure 2. Bubble plot of the relative abundance of bacterial 16S rRNA genes (%) in 28 cold seep sediment samples. Sample names consist of the abbreviation of the site name and sampling depth range in meters below seafloor (mbsf).

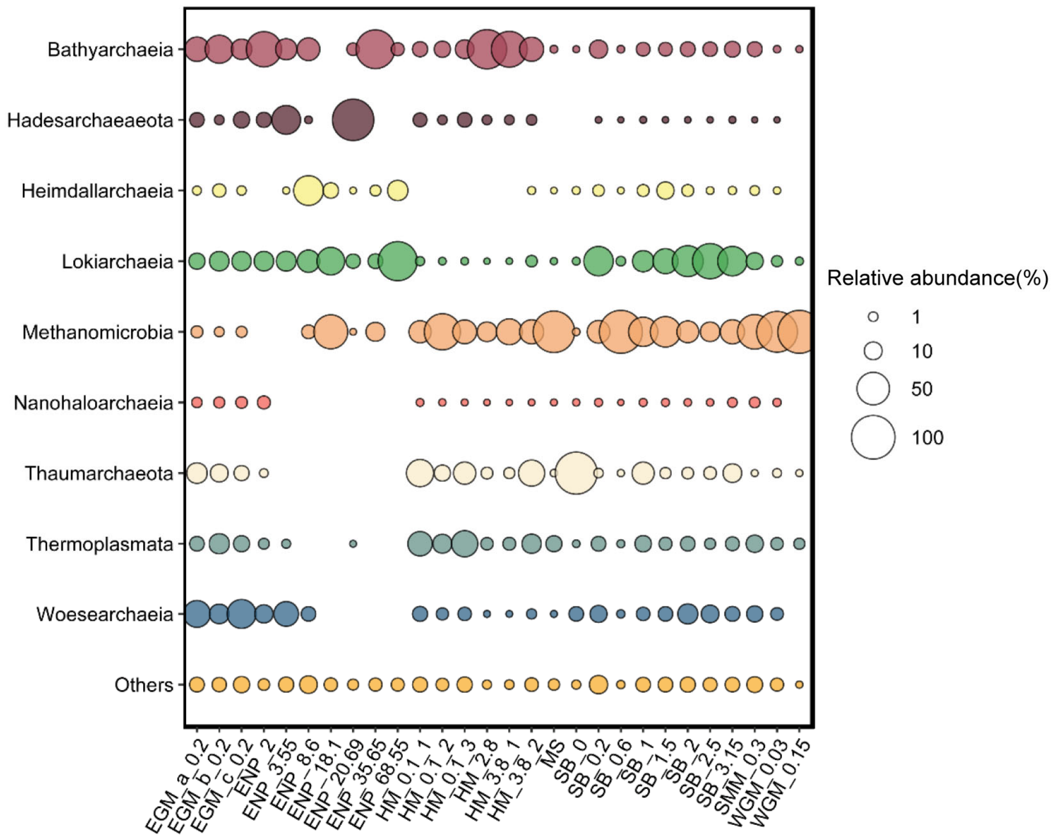

Supplementary Figure 3. Bubble plot of the relative abundance of archaeal 16S rRNA genes (%) in 28 cold seep sediment samples. Sample names consist of the abbreviation of the site name and sampling depth range in meters below seafloor (mbsf).

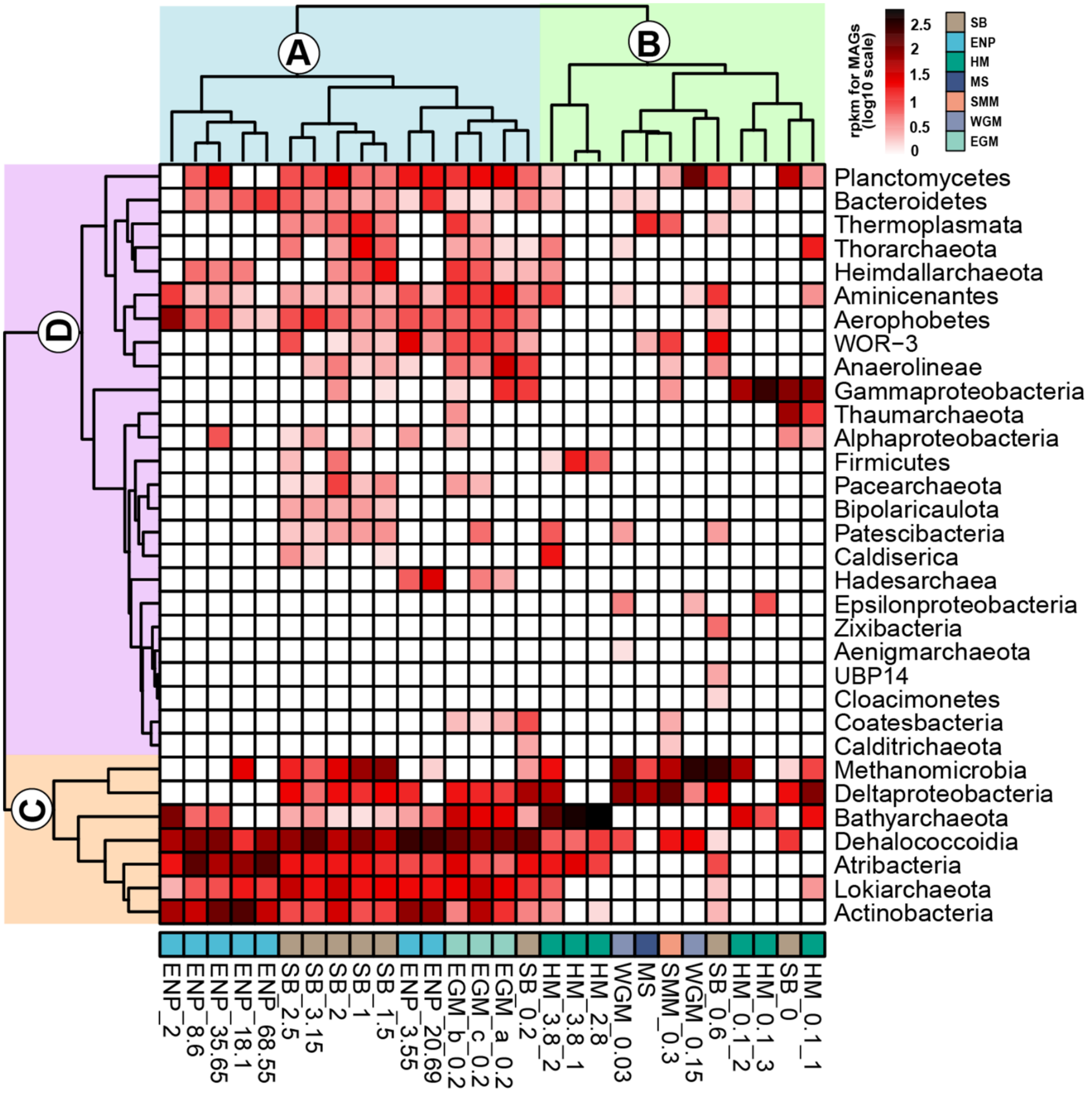

Supplementary Figure 4. Relative abundance of MAGs in different samples. The relative abundance was summed and log10 transformed at the phylum level (class level for Proteobacteria and Euryarchaeota). RPKM means reads per kilobase, per million mapped reads. They were clustered using Euclidean distance and Ward.D2 hierarchical clustering. A and B denote clustering of samples from different regions and depths; C and D denote clustering of MAGs from different taxonomies.

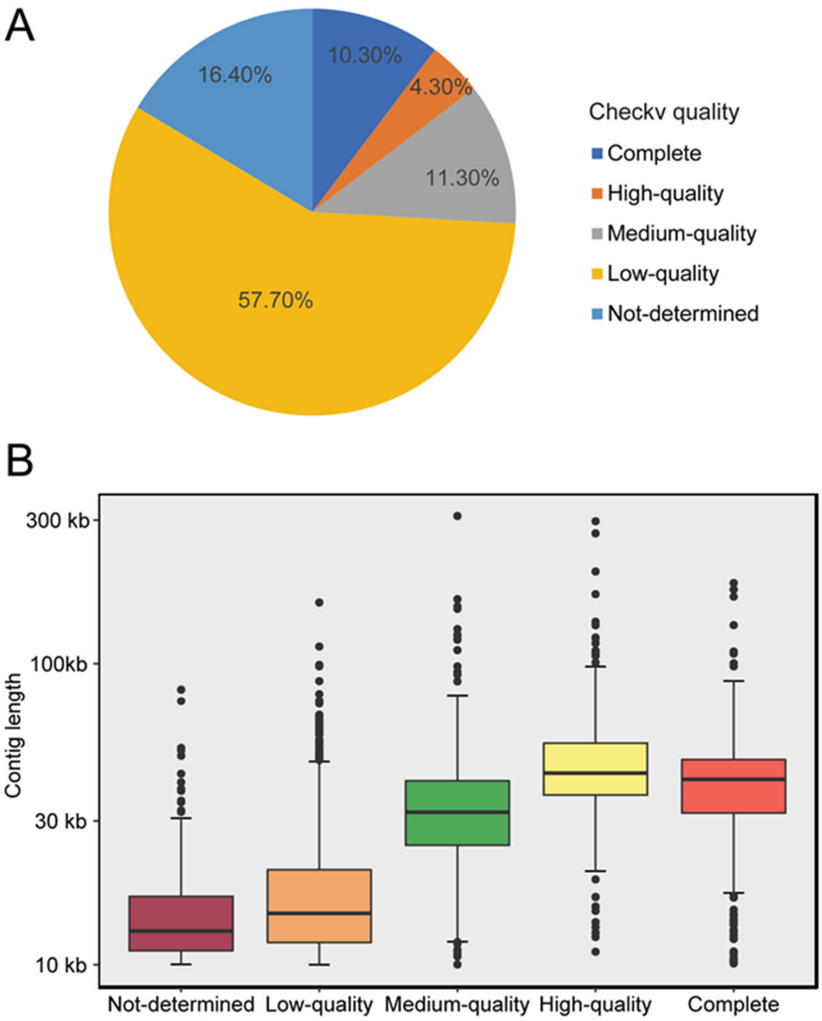

Supplementary Figure 5. Genome quality and completeness of vOTUs. (a) Proportion of genome quality tiers of cold seep viral populations. (b) Distribution of cold seep viral genomes across quality tiers. Quality of viral genomes were evaluated via CheckV based on estimated genome completeness. Complete: 100% completeness; high-quality: ≥ 90% completeness; medium-quality: 50-90% completeness; low-quality: < 50% completeness; not-determined: undetermined completeness.

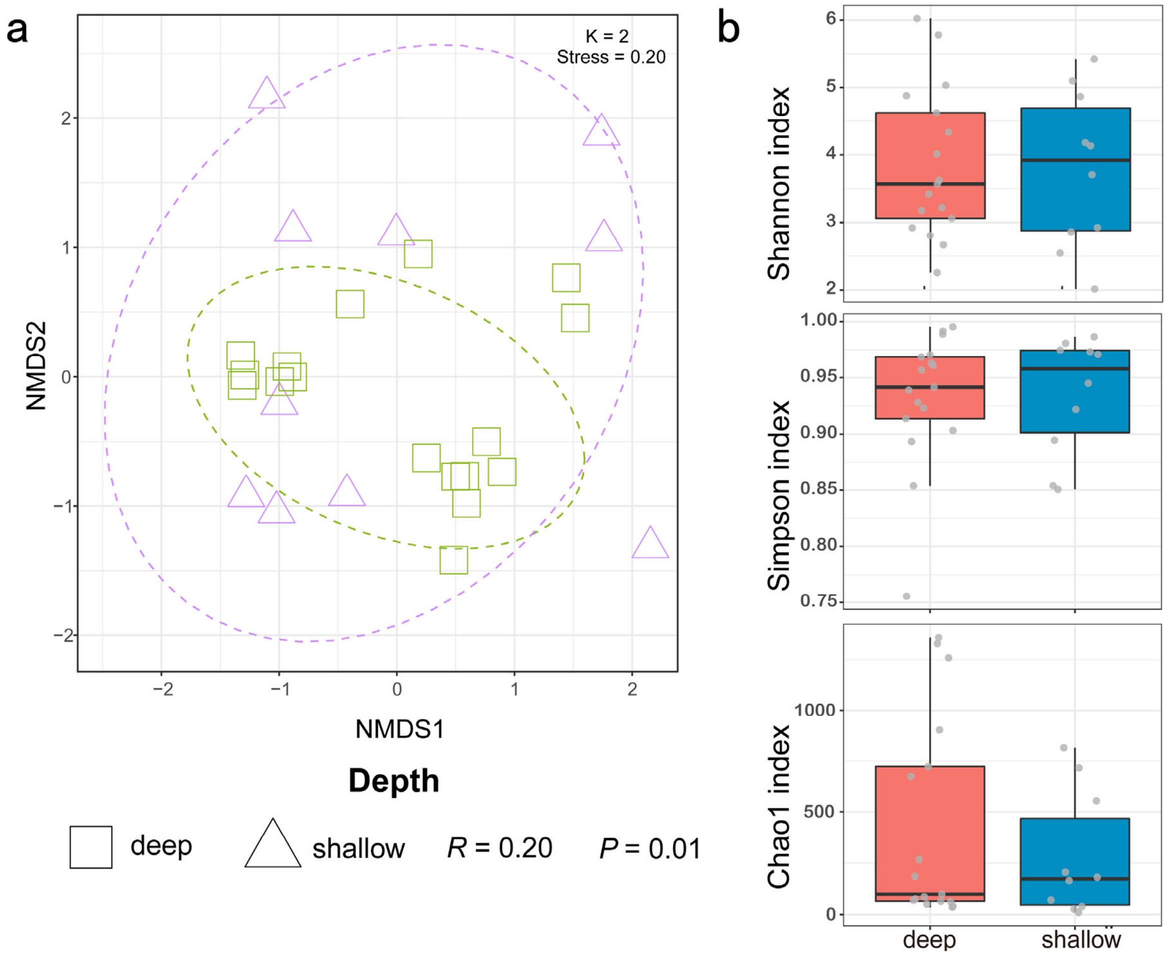

Supplementary Figure 6. Comparison of viral community diversity between shallow (0-0.2 mbsf) and deep (>0.2 mbsf) depths. Note that samples from Site MS (Mediterranean Sea, Amon mud volcano) were removed for this grouping due to lack of depths information. (a) NMDS analysis of a Bray-Curtis dissimilarity matrix calculated from RPKM values of vOTUs. ANOSIM was applied to test for the difference between viral communities between shallow and deep depths. (b) Shannon, Simpson and Chao1 indices of the viral community diversity from between shallow and deep depths. No significance was observed for these comparisons.

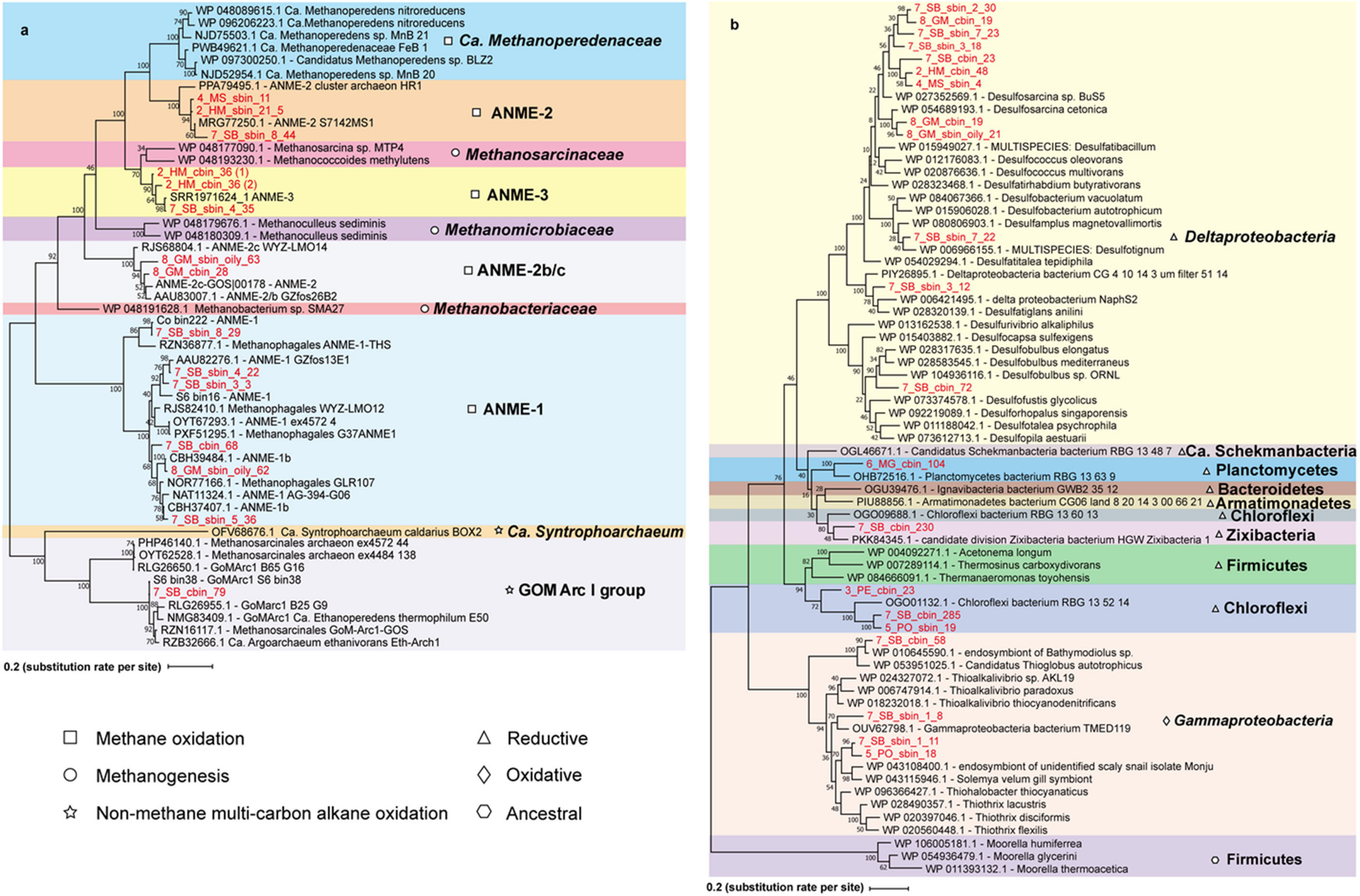
a
WP 048089615.1 Ca. Methanoperedens nitroreducens
WP 096206223.1 Ca.Methanoperedens nitroreducens
NJD75503.1 Ca. Methanoperedens sp. MnB 21
PWB49621.1 Ca. Methanoperedenaceae FeB 1
WP 097300250.1 - Candidatus Methanoperedens sp. BLZ2
NJD52954.1 Ca. Methanoperedens sp. MnB 20
Ca. Methanoperedenaceae
PPA79495.1 - ANME-2 cluster archaeon HR1
4_MS_sbin_11
2_HM_sbin_21_5
MRG77250.1 - ANME-2 S7142MS1
7_SB_sbin_8_44
ANME-2
WP 048177090.1 - Methanosarcina sp. MTP4
WP 048193230.1 - Methanococcoides methylutens
Methanosarcinaceae
2_HM_cbin_36 (1)
2_HM_cbin_36 (2)
SRR1971624_1 ANME-3
7_SB_sbin_4_35
ANME-3
WP 048179676.1 - Methanoculleus sediminis
WP 048180309.1 - Methanoculleus sediminis
Methanomicrobiaceae
RJS68804.1 - ANME-2c WYZ-LMO14
8_GM_sbin_oily_63
8_GM_cbin_28
ANME-2c-GOS|00178 - ANME-2
AAU83007.1 - ANME-2/b GZfos26B2
ANME-2b/c
WP 048191628.1 Methanobacterium sp. SMA27
Methanobacteriaceae
Co bin222 - ANME-1
7_SB_sbin_8_29
RZN36877.1 - Methanophagales ANME-1-THS
AAU82276.1 - ANME-1 GZfos13E1
7_SB_sbin_4_22
7_SB_sbin_3_3
S6 bin16 - ANME-1
RJS82410.1 Methanophagales WYZ-LMO12
OYT67293.1 - ANME-1 ex4572 4
PXF51295.1 - Methanophagales G37ANME1
7_SB_cbin_68
CBH39484.1 - ANME-1b
8_GM_sbin_oily_62
NOR77166.1 - Methanophagales GLR107
NAT11324.1 - ANME-1 AG-394-G06
CBH37407.1 - ANME-1b
7_SB_sbin_5_36
ANME-1
OFV68676.1 Ca. Syntrophoarchaeum caldarius BOX2
Ca. Syntrophoarchaeum
PHP46140.1 - Methanosarcinales archaeon ex4572 44
OYT62528.1 - Methanosarcinales archaeon ex4484 138
RLG26650.1 - GoMArc1 B65 G16
S6 bin38 - GoMArc1 S6 bin38
7_SB_cbin_79
RLG26955.1 - GoMarc1 B25 G9
NMG83409.1 - GoMArc1 Ca. Ethanoperedens thermophilum E50
RZN16117.1 - Methanosarcinales GoM-Arc1-GOS
RZB32666.1 Ca. Argoarchaeum ethanivorans Eth-Arch1
GOM Arc I group
0.2 (substitution rate per site)
b
7_SB_sbin_2_30
8_GM_cbin_19
7_SB_sbin_7_23
7_SB_sbin_3_18
7_SB_cbin_23
2_HM_cbin_48
4_MS_sbin_4
WP 027352569.1 - Desulfosarcina sp. BuS5
WP 054689193.1 - Desulfosarcina cetonica
8_GM_cbin_19
8_GM_sbin_oily_21
WP 015949027.1 - MULTISPECIES: Desulfatibacillum
WP 012176083.1 - Desulfococcus oleovorans
WP 020876636.1 - Desulfococcus multivorans
WP 028323468.1 - Desulfatirhabdium butyrativorans
WP 084067366.1 - Desulfobacterium vacuolatum
WP 015906028.1 - Desulfobacterium autotrophicum
WP 080806903.1 - Desulfamplus magnetovallimortis
7_SB_sbin_7_22
WP 006966155.1 - MULTISPECIES: Desulfotignum
WP 054029294.1 - Desulfatitalea tepidiphila
PIY26895.1 - Deltaproteobacteria bacterium CG 4 10 14 3 um filter 51 14
7_SB_sbin_3_12
WP 006421495.1 - delta proteobacterium NaphS2
WP 028320139.1 - Desulfatiglans anilini
WP 013162538.1 - Desulfurivibrio alkaliphilus
WP 015403882.1 - Desulfocapsa sulfexigens
WP 028317635.1 - Desulfobulbus elongatus
WP 028583545.1 - Desulfobulbus mediterraneus
WP 104936116.1 - Desulfobulbus sp. ORNL
7_SB_cbin_72
WP 073374578.1 - Desulfofustis glycolicus
WP 092219089.1 - Desulforhopalus singaporensis
WP 011188042.1 - Desulfotalea psychrophila
WP 073612713.1 - Desulfopila aestuarii
Deltaproteobacteria
OGL46671.1 - Candidatus Schekmanbacteria bacterium RBG 13 48 7
Ca. Schekmanbacteria
6_MG_cbin_104
OHB72516.1 - Planctomycetes bacterium RBG 13 63 9
Planctomycetes
OGU39476.1 - Ignavibacteria bacterium GWB2 35 12
Bacteroidetes
PIU88856.1 - Armatimonadetes bacterium CG06 land 8 20 14 3 00 66 21
Armatimonadetes
OGO09688.1 - Chloroflexi bacterium RBG 13 60 13
Chloroflexi
7_SB_cbin_230
PKK84345.1 - candidate division Zixibacteria bacterium HGW Zixibacteria 1
Zixibacteria
WP 004092271.1 - Acetonema longum
WP 007289114.1 - Thermosinus carboxydivorans
WP 084666091.1 - Thermanaeromonas toyohensis
Firmicutes
3_PE_cbin_23
OGO01132.1 - Chloroflexi bacterium RBG 13 52 14
7_SB_cbin_285
5_PO_sbin_19
Chloroflexi
7_SB_cbin_58
WP 010645590.1 - endosymbiont of Bathymodiolus sp.
WP 053951025.1 - Candidatus Thioglobus autotrophicus
WP 024327072.1 - Thioalkalivibrio sp. AKL19
WP 006747914.1 - Thioalkalivibrio paradoxus
WP 018232018.1 - Thioalkalivibrio thiocyanodenitrificans
7_SB_sbin_1_8
OUV62798.1 - Gammaproteobacteria bacterium TMED119
7_SB_sbin_1_11
5_PO_sbin_18
WP 043108400.1 - endosymbiont of unidentified scaly snail isolate Monju
WP 043115946.1 - Solemya velum gill symbiont
WP 096366427.1 - Thiohalobacter thiocyanaticus
WP 028490357.1 - Thiothrix lacustris
WP 020397046.1 - Thiothrix disciformis
WP 020560448.1 - Thiothrix flexilis
Gammaproteobacteria
WP 106005181.1 - Moorella humiferrea
WP 054936479.1 - Moorella glycerini
WP 011393132.1 - Moorella thermoacetica
Firmicutes
0.2 (substitution rate per site)
Methane oxidation
Methanogenesis
Non-methane multi-carbon alkane oxidation
Reductive
Oxidative
Ancestral

Supplementary Figure 7. Maximum likelihood phylogenetic trees of methyl-Coenzyme M reductase and dissimilatory sulfite reductase. Phylogenetic tree constructed based on alignments of amino acid sequences of (a) McrA and (b) DsrA genes. Bootstrap values are indicated as numbers. Genes of putative hosts in this study are highlighted in red.

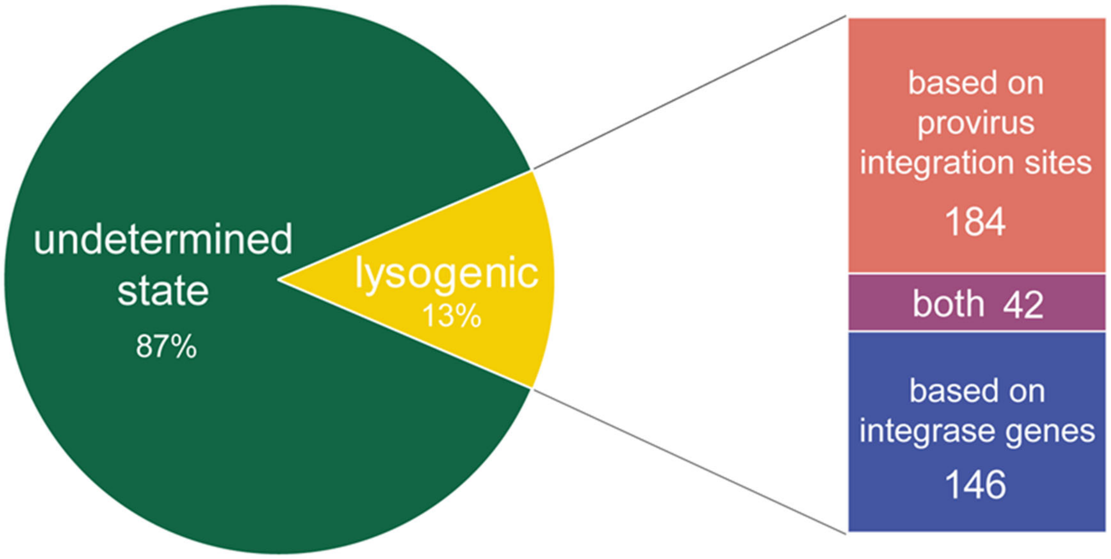

Supplementary Figure 8. Proportion of putatively lysogenic viruses of all the cold seep vOTUs. Lysogenic viruses were identified based on provirus integration sites and integrase genes using CheckV and VIBRANT.
